# Supplementary figures and images for: Smartphone Postural Sway and Pronator Drift tests as Measures of Neurological Disability
Source: medRxiv. 2024 Nov 21:2024.11.20.24317196. Preprint. [Version 1] doi: 10.1101/2024.11.20.24317196 (PMC11722448; doi:10.1101/2024.11.20.24317196)

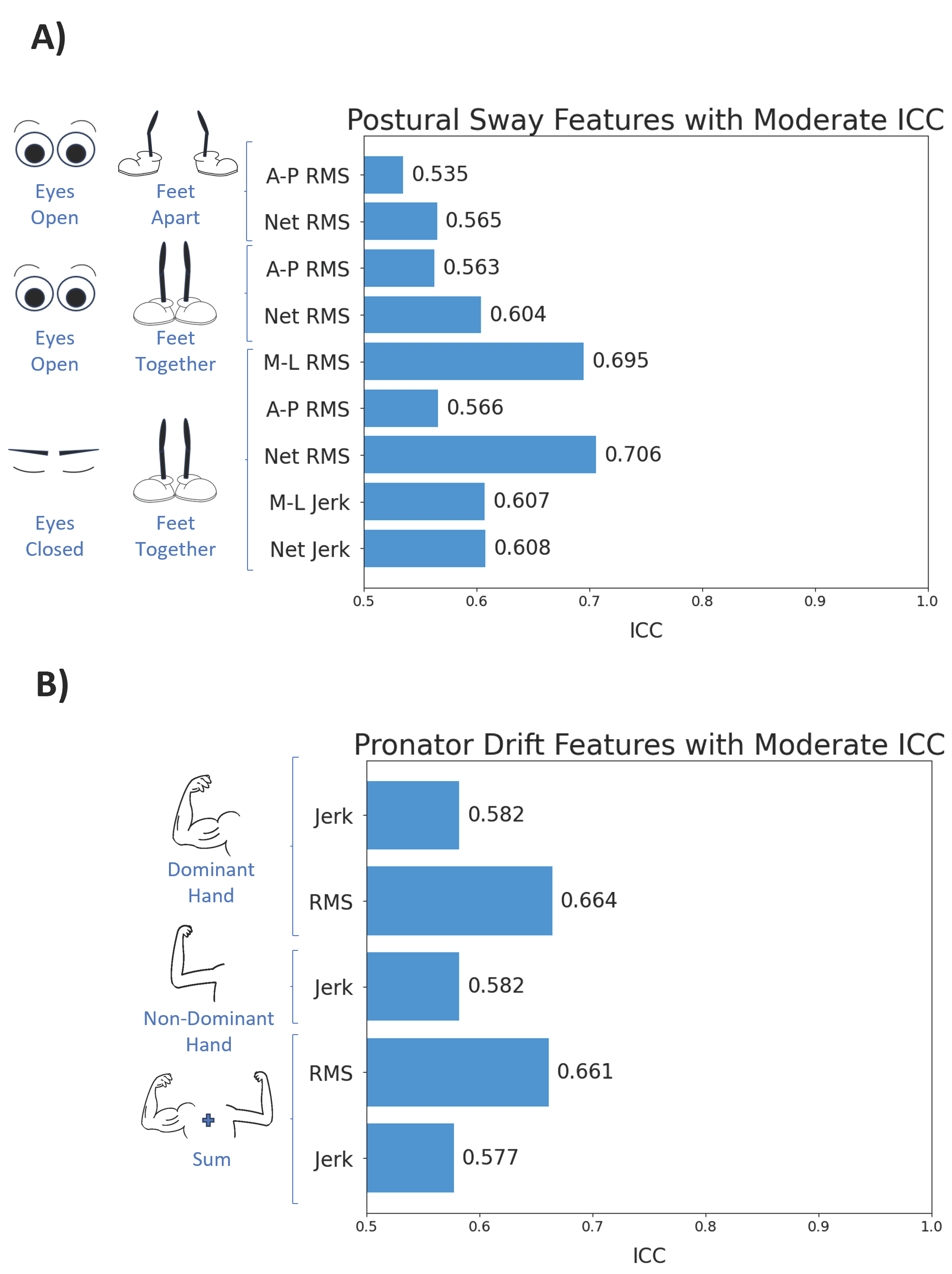

Supplement: Supplement 22 [file media-22.tif]

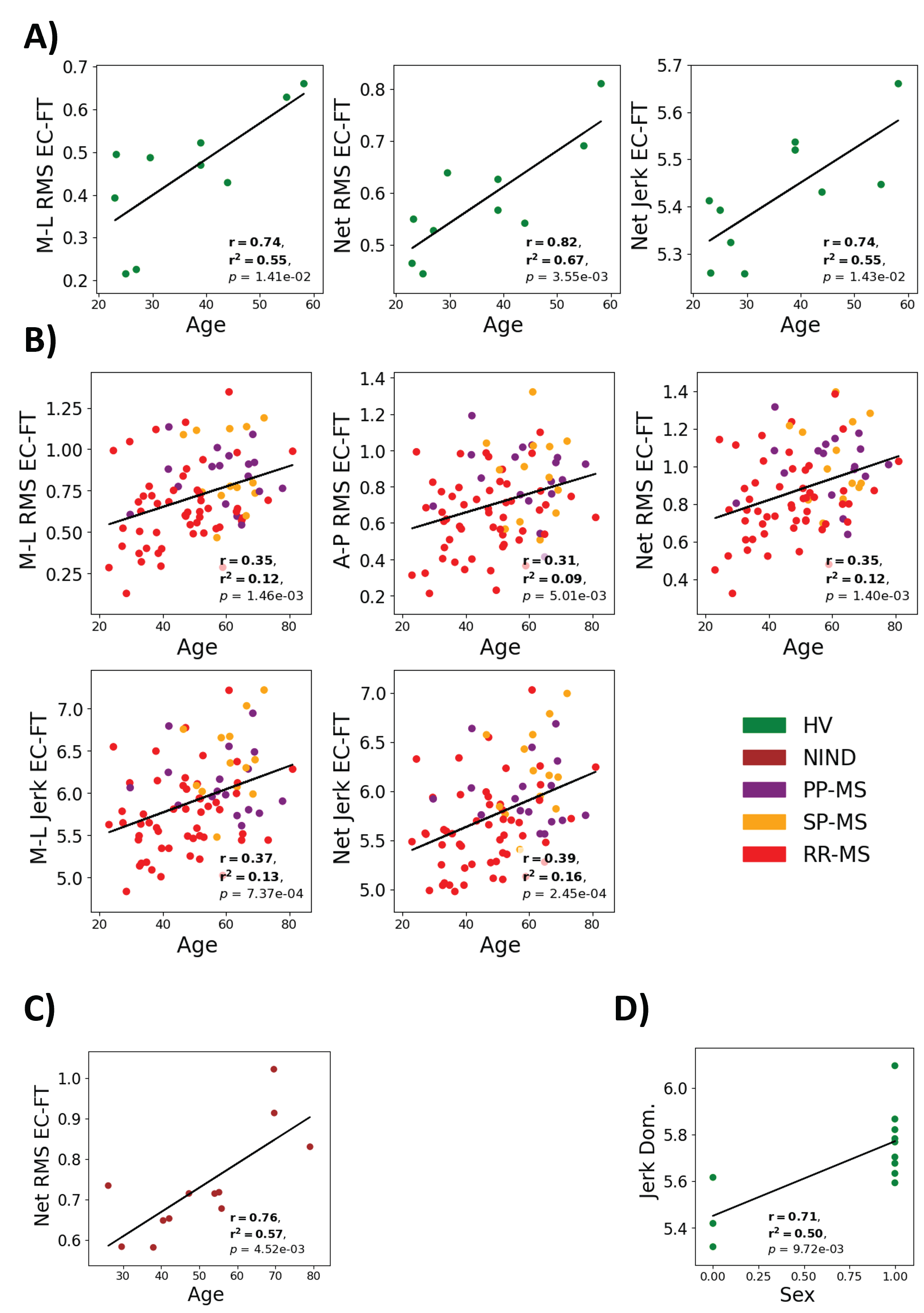

Supplement: Supplement 23 [file media-23.tif]

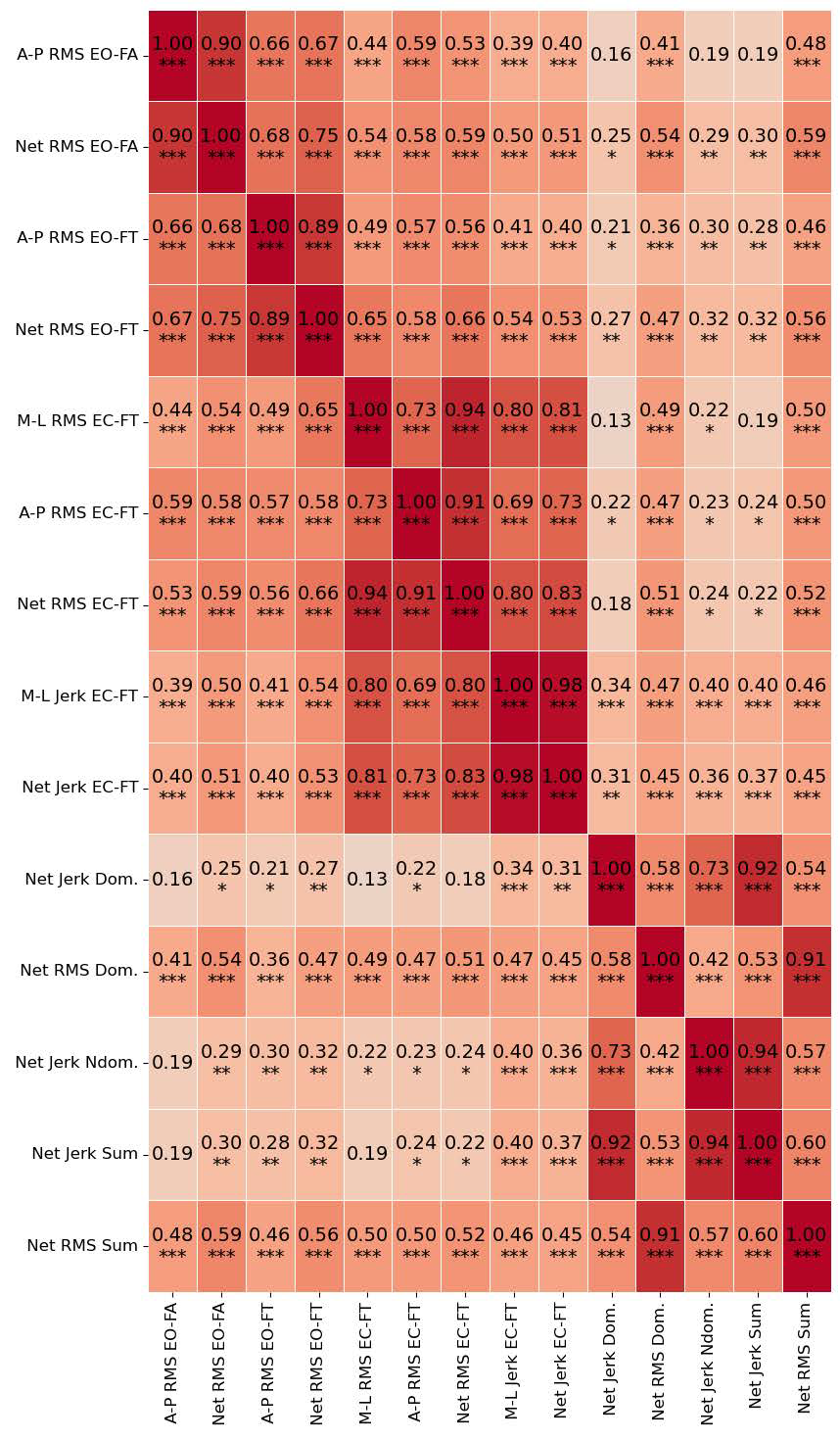

Supplement: Supplement 24 [file media-24.tif]

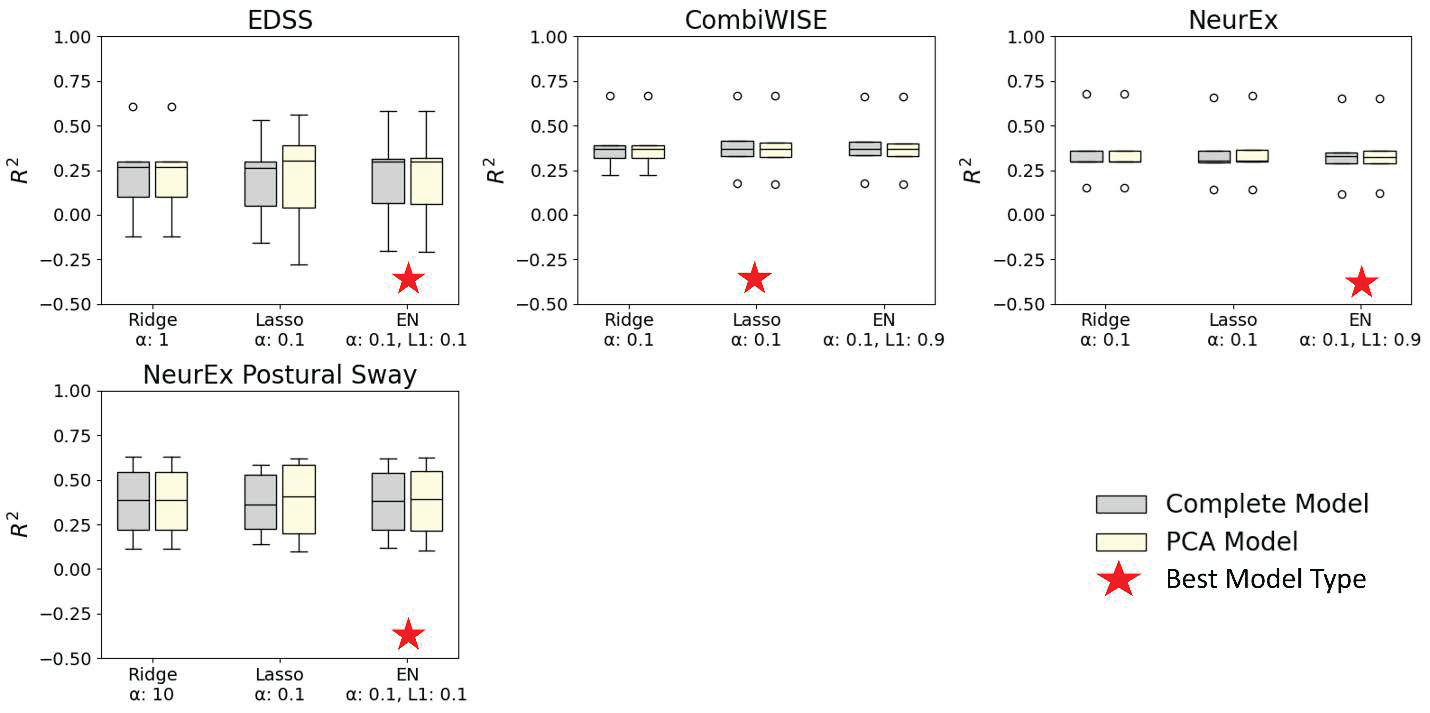

Supplement: Supplement 25 [file media-25.tif]

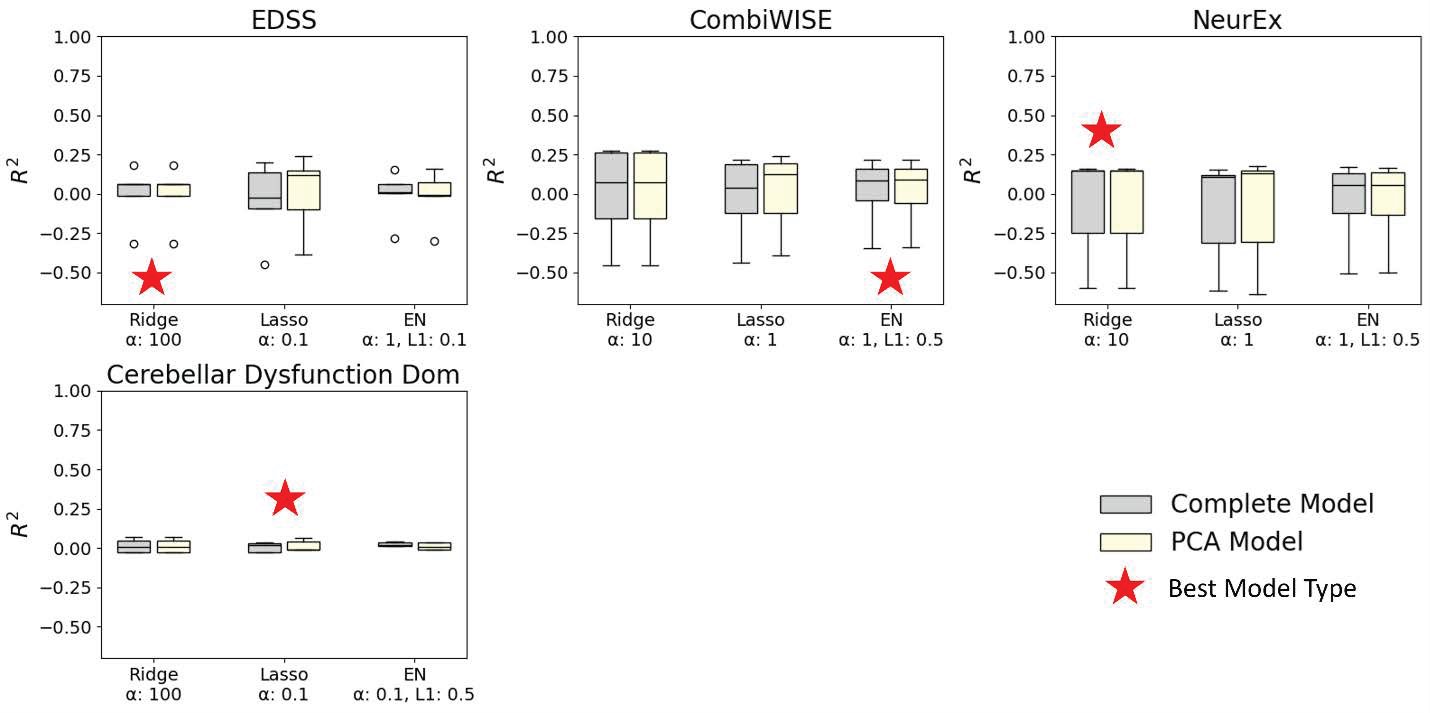

Supplement: Supplement 26 [file media-26.tif]

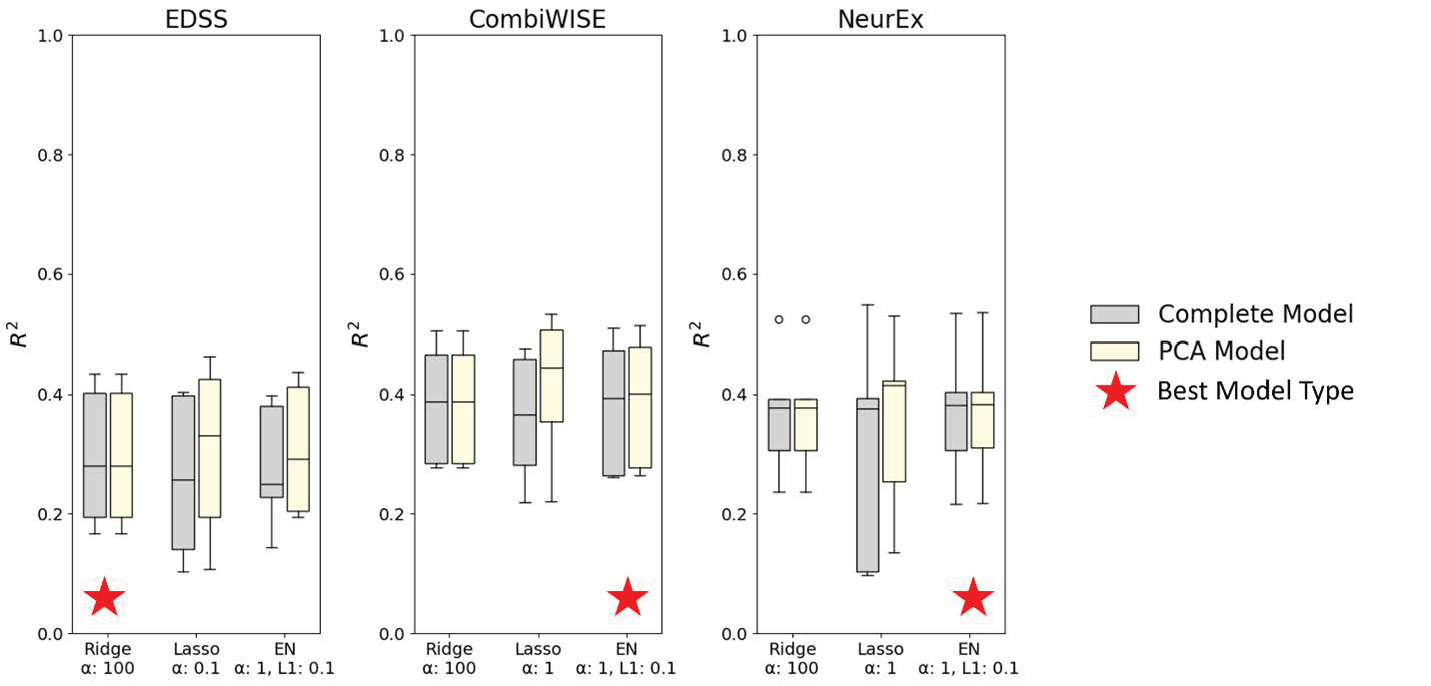

Supplement: Supplement 27 [file media-27.tif]

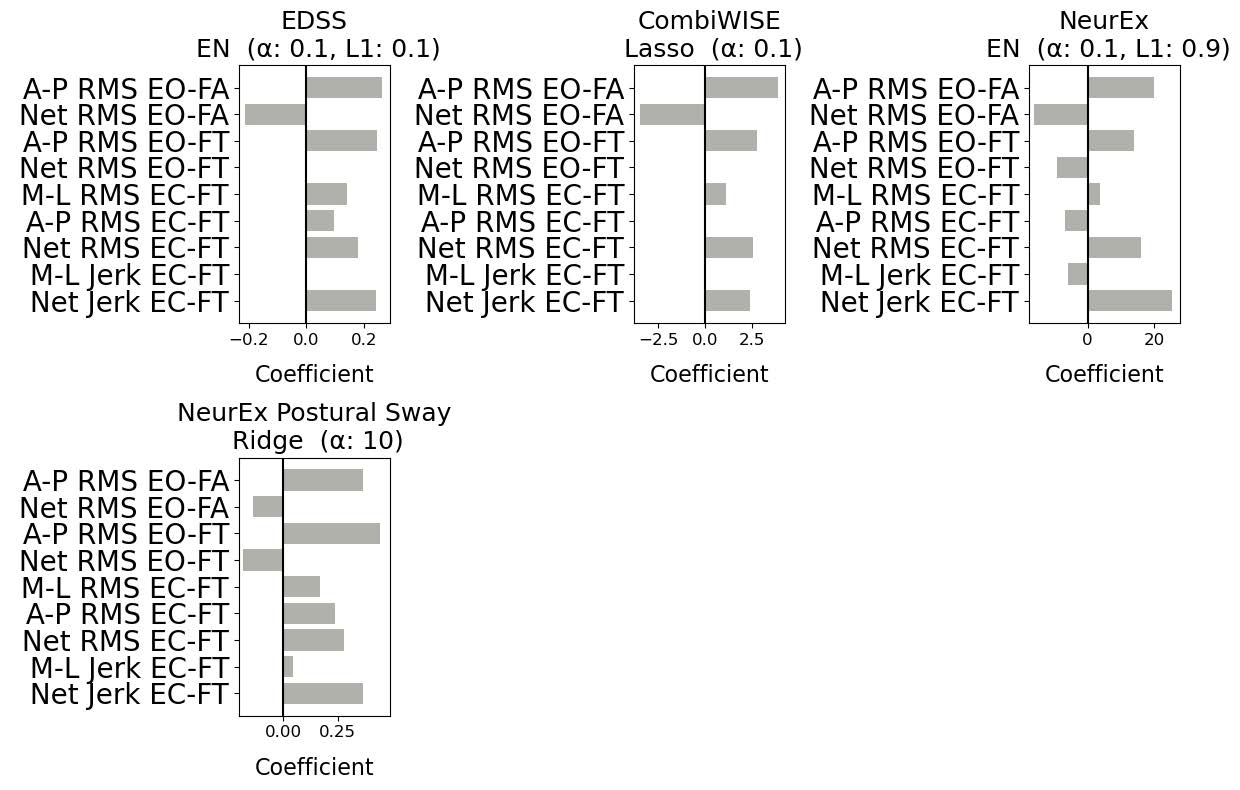

Supplement: Supplement 28 [file media-28.tif]

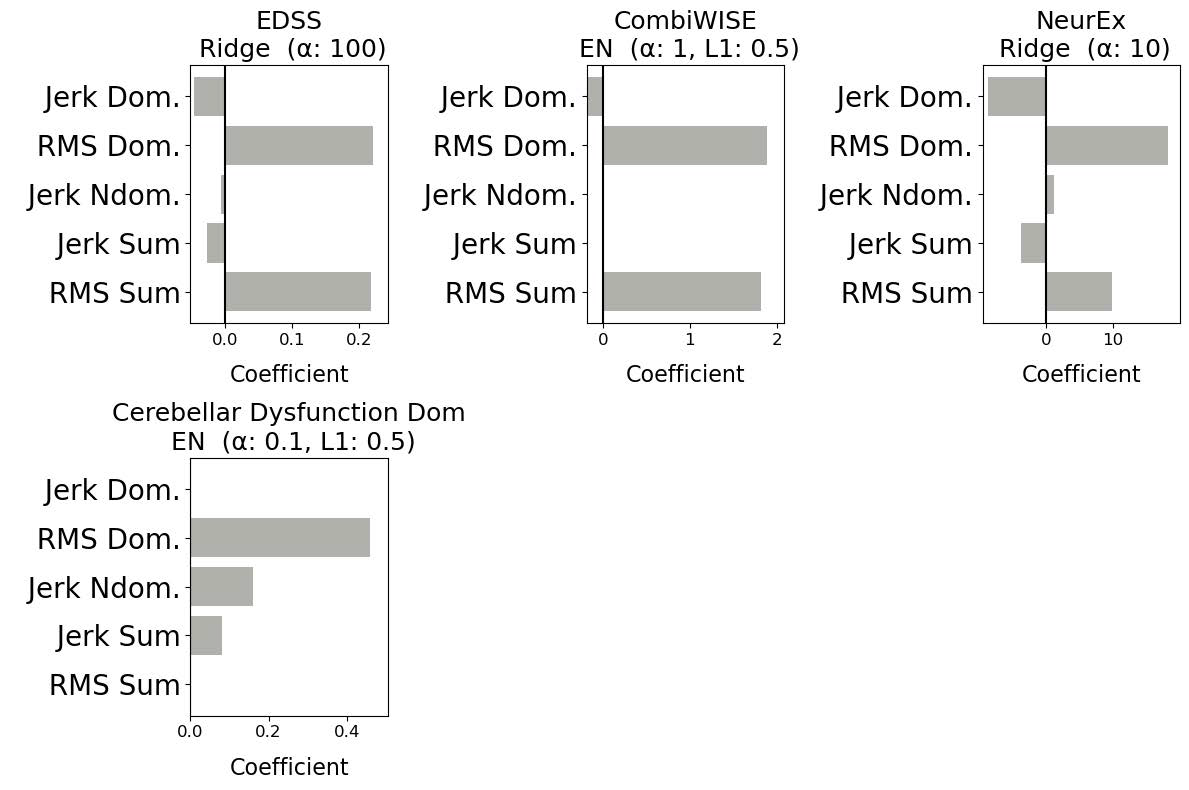

Supplement: Supplement 29 [file media-29.tif]

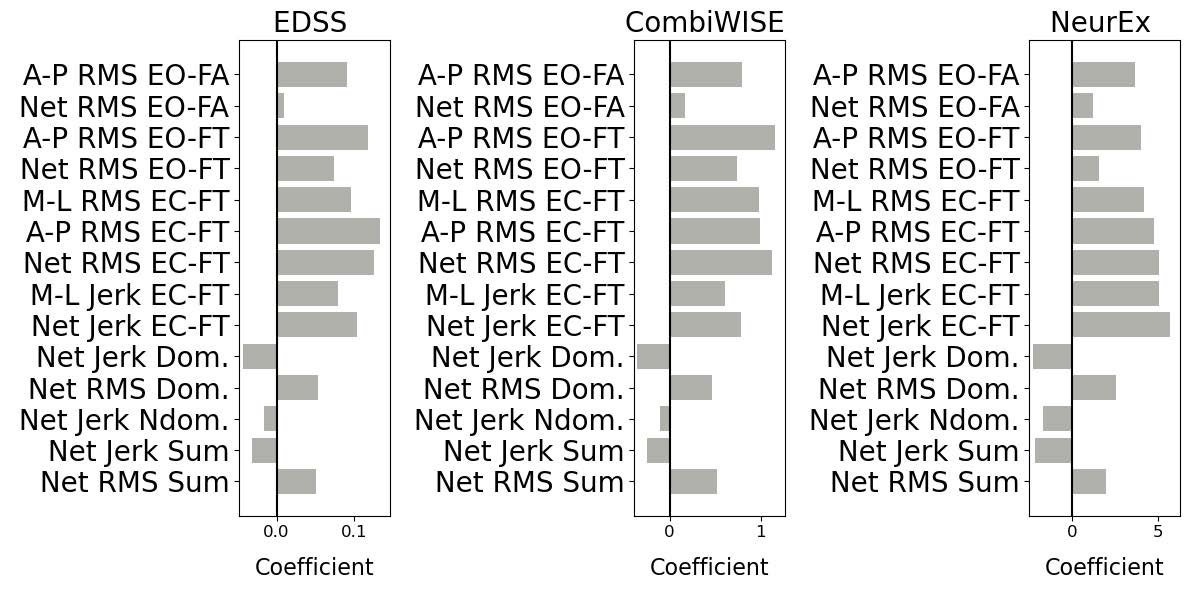

Supplement: Supplement 30 [file media-30.tif]

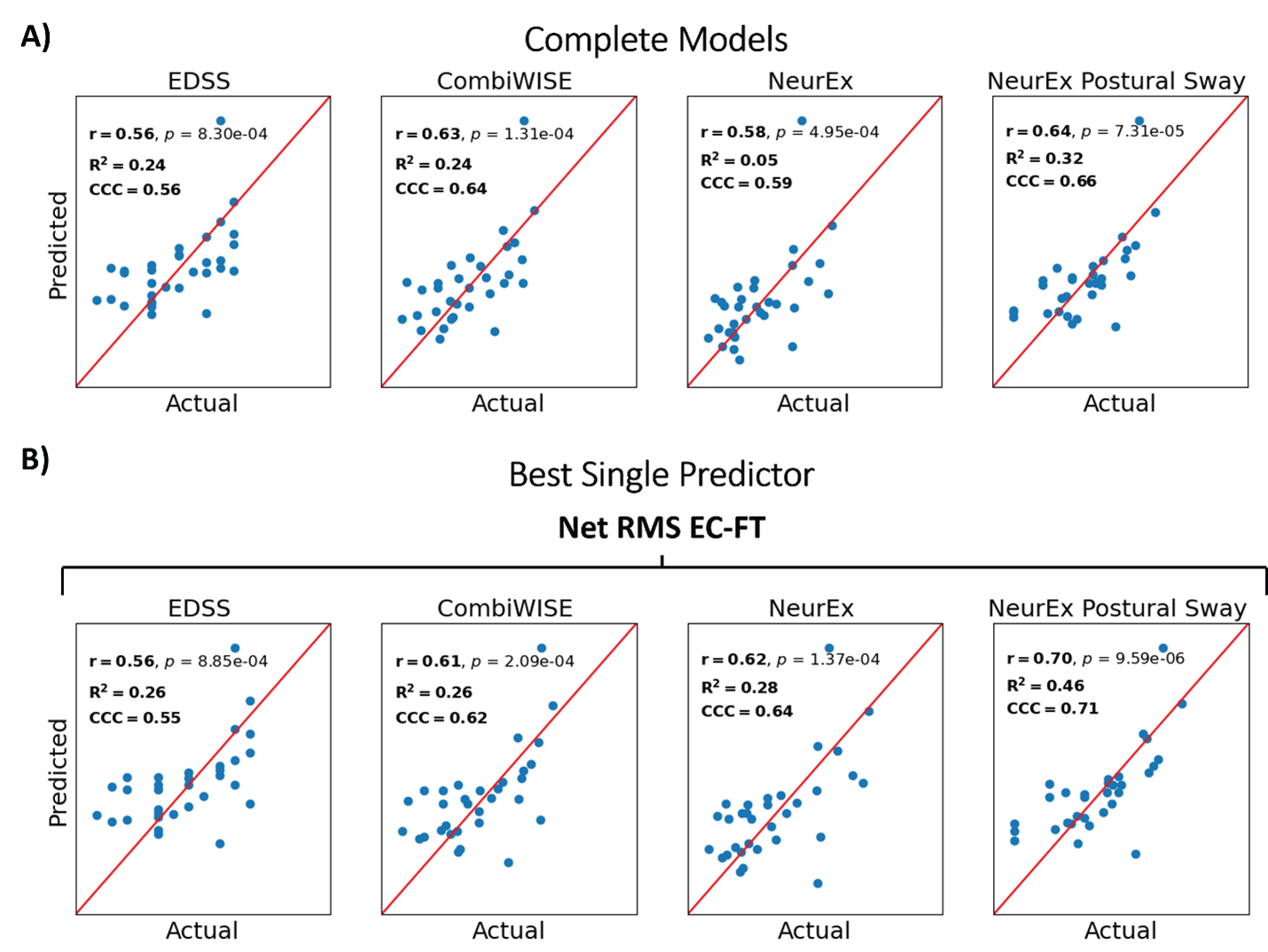

Supplement: Supplement 31 [file media-31.tif]

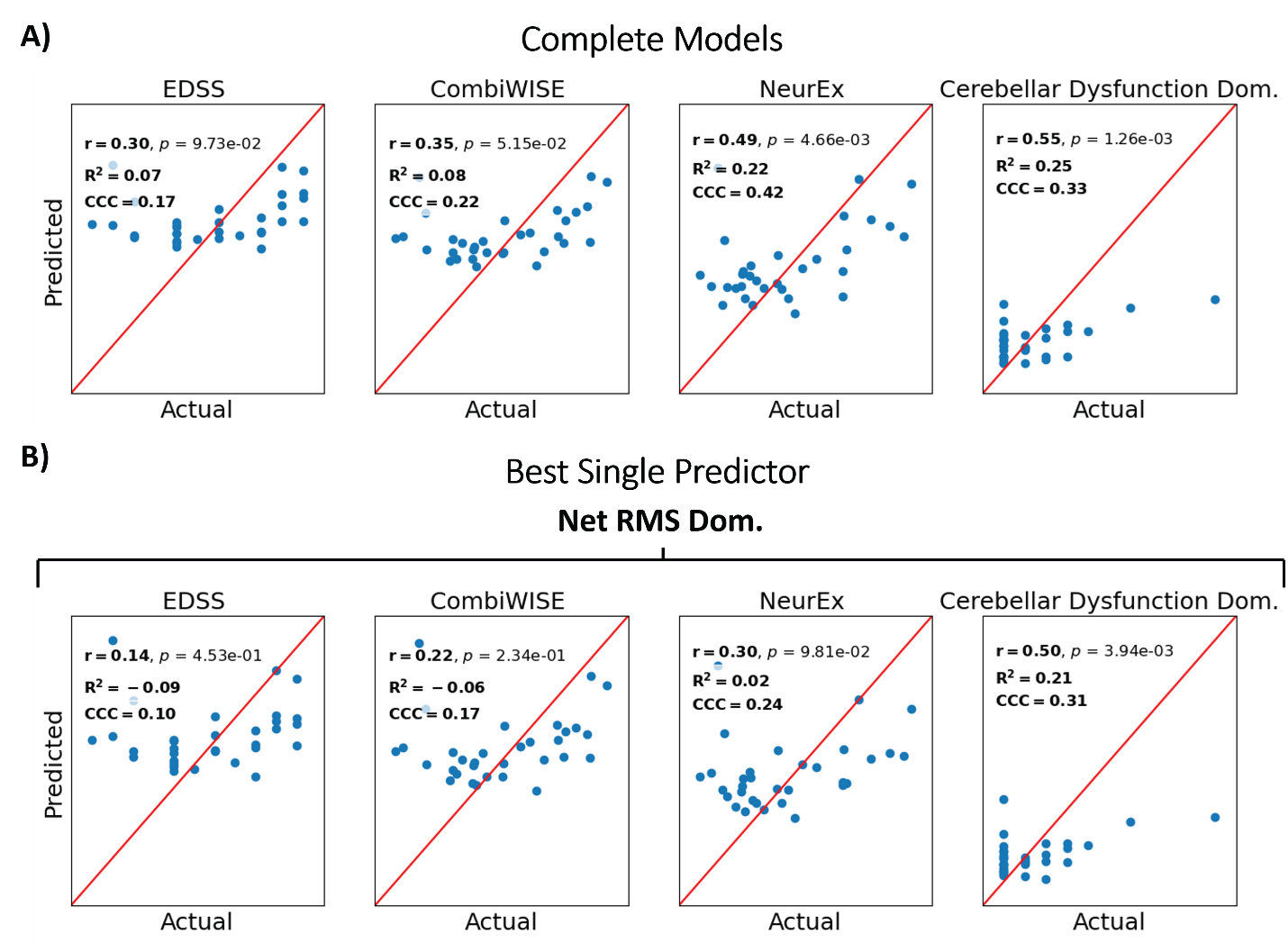

Supplement: Supplement 32 [file media-32.tif]

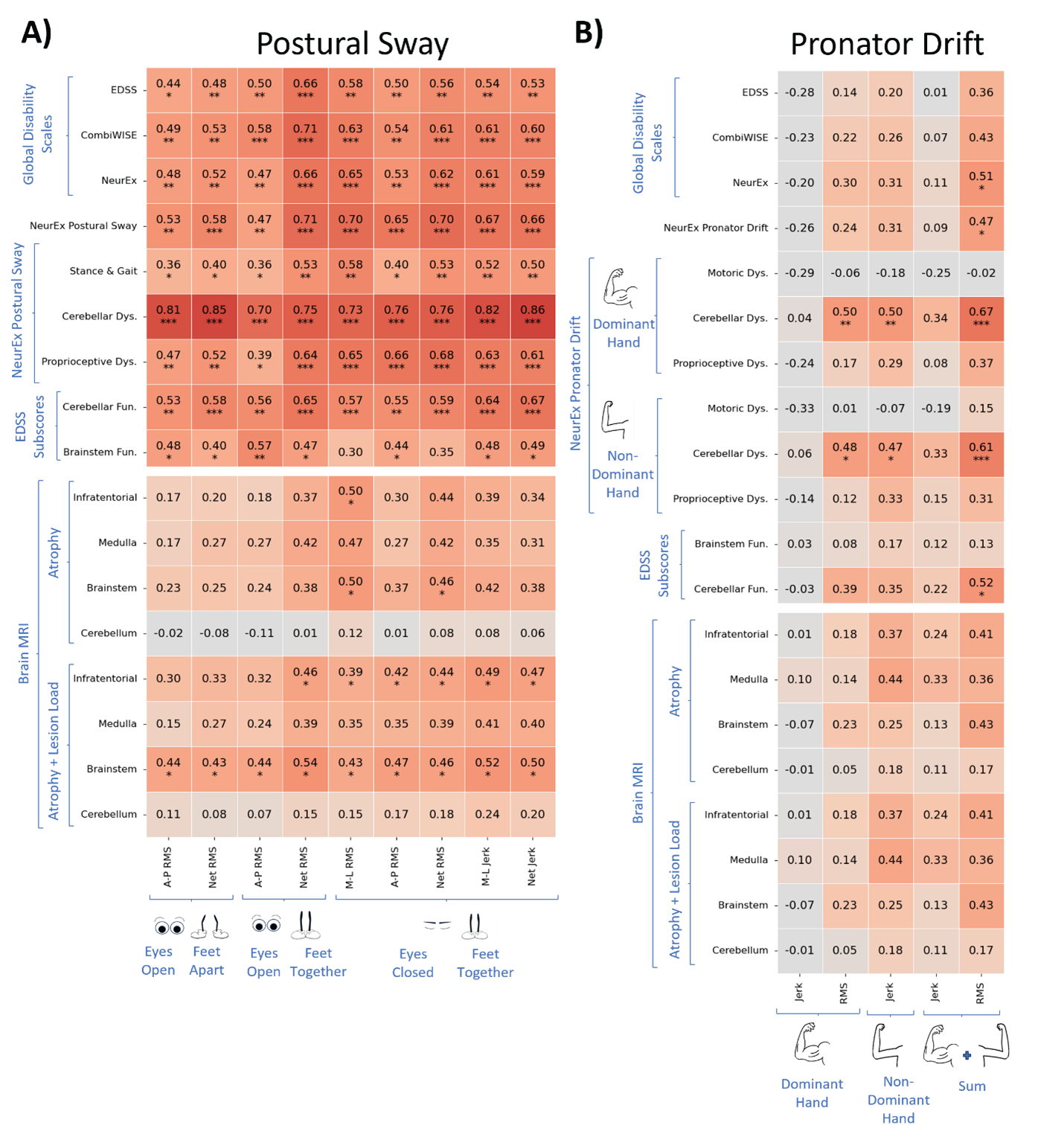

Supplement: Supplement 33 [file media-33.tif]
